# Sunday for the People

**Published:** 1891-11

**Authors:** 


### SUNDAY FOR THE PEOPLE.

Mrs. Isabella Beecher Hooker, at the meeting of the Board of Lady Managers of the World's Fair in Chicago, on the 5th inst., said in the discussion on Sunday opening : If I were an autocrat I'd open the Fair every day as early as I could wake up, though I'm not an early riser, but I would close every grog shop within twenty miles of Chicago. Sunday I would open all the art buildings and all the buildings where there is music, turn the whole place into a great Sunday-school, and notify the nations of the earth to come early in order to get into the Sunday-school. The greatest display of the whole Fair should be the display of the spirit expressed in the commendment, "Thou shall love thy neighbor as thyself." We earnestly command the sentiments of Mrs. Hooker to the World's Fair managers. It is time that our law-makers awoke to the modern idea that the seventh day of rest is for the toiling masses, no less than for the fashionables whose hours of employment are few, and whose diversions are many. To the hardy mechanic and workman rest is recreation, and there are no better Sundays ermons than those to be had in exhibition of human progress in art, in science, and in morals.

